# Efficacy and safety of probiotics combined with bismuth-containing quadruple therapy for *Helicobacter pylori* eradication: a systematic review and meta-analysis

**DOI:** 10.3389/fmicb.2025.1704508

**Published:** 2026-01-07

**Authors:** Shunhua Zhou, Yunfeng Yu, Jiaxuan Tian, Xiaojuan Wang, Meiyan Zeng, Houpan Song

**Affiliations:** 1 Hunan Provincial Key Laboratory of Traditional Chinese Medicine Diagnostics, Hunan University of Chinese Medicine, Changsha, China; 2 School of Traditional Chinese Medicine, Hunan University of Chinese Medicine, Changsha, China

**Keywords:** *Helicobacter pylori*, probiotic, bismuth-containing quadruple therapy, systematic review, meta-analysis

## Abstract

**Objective:**

*Helicobacter pylori* (*H. pylori*) infection represents a global health challenge. This study aimed to evaluate the effect of probiotic supplementation on the efficacy and safety of bismuth-containing quadruple therapy (BQT) for *H. pylori* eradication.

**Methods:**

Randomized controlled trials (RCTs) meeting the eligibility criteria were identified through systematic searches of five databases, including PubMed, Embase, Cochrane Library, Web of Science, and ClinicalTrials.gov. Meta-analyses were conducted using Review Manager software with a random-effects model to calculate pooled relative risks (RR) and 95% confidence intervals (CI). The potential publication bias was evaluated qualitatively, and the certainty of the evidence was subsequently assessed.

**Results:**

In total, 10 RCTs involving 1,630 patients were included in the analysis. The results demonstrated that, compared with the BQT group, the *H. pylori* eradication rate was significantly higher in the probiotics combined with the BQT group (RR 1.06, 95% CI 1.01–1.11, *p* = 0.009, ARR = 7.5%, NNT ≈ 13). Additionally, the combined therapy significantly reduced the adverse event rate (RR 0.58, 95% CI 0.42–0.80, *p* = 0.001, ARR = 11.1%, NNT ≈ 9), diarrhea (RR 0.48, 95% CI 0.32–0.73, *p* = 0.0007, ARR = 0.7%, NNT ≈ 142), and constipation (RR 0.53, 95% CI 0.29–0.94, *p* = 0.04, ARR = 2.5%, NNT ≈ 40). However, no statistically significant differences were observed for other specific adverse events, including nausea, vomiting, anorexia, heartburn, belching, taste disturbance, abdominal pain, and abdominal bloating. Sensitivity analyses confirmed the robustness of the results for the *H. pylori* eradication rate and adverse event rate.

**Conclusion:**

Combining probiotics with BQT significantly increases the *H. pylori* eradication rate and reduces adverse events, particularly diarrhea and constipation. These findings support the role of probiotics as a supplementary strategy to enhance both the efficacy and safety of *H. pylori* eradication therapy. However, the overall certainty of the evidence is low to very low, and the optimal probiotic protocol has yet to be determined, highlighting the need for further high-quality research.

**Systematic review registration:**

https://www.crd.york.ac.uk/PROSPERO/view/CRD420251060806, Identifier CRD420251060806.

## 1 Introduction

*Helicobacter pylori* (*H. pylori*) is a Gram-negative pathogenic bacterium, and its infection has become a global public health issue, affecting approximately half of the world’s population (Bi et al., 2022). Epidemiological evidence shows that *H. pylori* infection mainly occurs in early childhood (usually before the age of 10). Through persistent colonization of the gastric mucosa and induction of chronic inflammation, this pathogen has been confirmed to be etiologically associated with a variety of gastric diseases, including peptic ulcers, gastric adenocarcinoma, and precancerous lesions such as atrophic gastritis (Cai et al., 2019; Liou et al., 2020; Malfertheiner et al., 2017). International consensus recommends that all individuals infected with *H. pylori* should receive eradication therapy unless there are clear contraindications (Sugano et al., 2015). In regions with low clarithromycin resistance rates, the first-line eradication regimen is recommended to be triple therapy, which consists of a proton pump inhibitor (PPI), clarithromycin combined with either amoxicillin or metronidazole (Malfertheiner et al., 2017). However, with the increasing drug resistance of *H. pylori* (Suzuki et al., 2020), the eradication rate of standard triple therapy has significantly decreased from over 90% to below 60%. Against this background, guidelines recommend bismuth-containing or bismuth-free quadruple therapy as the preferred empirical treatment regimen (Fallone et al., 2019). As a low-toxicity “green” metal, bismuth is widely used in the pharmaceutical field for treating various diseases and also plays an important role in the eradication of *H. pylori* (Frei et al., 2020). The first-line regimen recommended in current Chinese guidelines is bismuth-containing quadruple therapy (BQT), which consists of a PPI, a bismuth agent, and two antibiotics. This regimen exhibits high-efficiency eradication ability against both sensitive strains and drug-resistant strains of *H. pylori*, with an eradication rate of up to 90% (Yin et al., 2023), and can be used to overcome drug resistance and improve eradication efficacy. However, caution is needed regarding the potential adverse events associated with BQT, such as headache, diarrhea, constipation, abdominal pain, abdominal bloating, and nausea (Yang et al., 2020). These adverse events are common causes of reduced patient compliance, which in turn leads to eradication failure (Viazis et al., 2022). Therefore, it is necessary to explore an adjuvant strategy that improves the *H. pylori* eradication rate and reduces adverse events, so as to maximize the improvement of the clinical application status of BQT.

The gastrointestinal tract harbors a diverse array of microorganisms, which are crucial for maintaining the balance of the gastrointestinal flora and overall health. However, *H. pylori* infection disrupts this balance, leading not only to inflammatory responses in the gastric mucosa (Pachathundikandi et al., 2020) but also to gastrointestinal dysbiosis in patients (Lou et al., 2007). Notably, a vicious cycle can form between inflammation and dysbiosis. For one, dysbiosis alters the gastric microenvironment, thereby enhancing *H. pylori* colonization; for another, chronic inflammation further disturbs the balance of the intestinal flora (Fabbri and Bracci, 2022). This means that while antibiotics are used to reduce inflammation, they indiscriminately kill gastrointestinal microorganisms, resulting in intestinal flora disturbance. In addition, the use of antibiotics may also significantly increase the risk of adverse reactions such as antibiotic-associated diarrhea. Against this background, probiotics have shown significant potential in regulating the balance of intestinal flora, enhancing immunity, and alleviating gastrointestinal adverse reactions (Hill et al., 2014). Studies have demonstrated that probiotic supplementation has been used to alleviate gastrointestinal diseases by restoring the bacterial microbiota through various mechanisms (Pochakom et al., 2022; Verna and Lucak, 2010). Early reports indicated that the eradication rate of single probiotic therapy was only 35%, which is insufficient for the effective eradication of *H. pylori* (Baryshnikova et al., 2023). However, subsequent studies have pointed out that combining probiotics with BQT can significantly improve the *H. pylori* eradication rate while reducing the adverse event rate (Keikha and Karbalaei, 2021; Mcfarland et al., 2016). These pieces of evidence support the positive role of probiotics in improving the gastrointestinal micro-environment and provide a basis for the therapeutic idea of using probiotics as an adjuvant to BQT for *H. pylori* eradication.

Although existing studies suggest that probiotics have a certain adjuvant therapeutic effect on the eradication of *H. pylori*, the current evidence mainly comes from single-center, small-sample randomized controlled trials (RCTs). These studies lack sufficient statistical power to comprehensively evaluate the efficacy and safety of the combined use of probiotics and BQT. Therefore, the general applicability and clinical promotability of the probiotic-combined BQT regimen remain unclear, and a comprehensive analysis of high-quality evidence is required to develop a standardized treatment protocol. This study aims to comprehensively assess the clinical value of the probiotic-combined BQT regimen in improving eradication rate and safety through a systematic review and meta-analysis, with the goal of providing evidence-based support for optimizing *H. pylori* eradication regimens in the era of drug resistance.

## 2 Methods

This study, which has been registered on the PROSPERO platform (CRD420251060806), is conducted in accordance with the current PRISMA 2020 guideline.

### 2.1 Data sources and search strategy

The systematic search was carried out across multiple electronic databases, such as PubMed, Embase, Cochrane Library, Web of Science, and ClinicalTrials.gov, to identify potentially eligible studies published before 30 April 2025. The search strategy utilized the following terms: (*Helicobacter pylori* OR *H. pylori* OR *Helicobacter nemestrinae* OR *Campylobacter pylori* OR *Campylobacter pyloridis*) AND (Probiotic OR Probiotics OR *Bacillus* OR *Bacillus bifida* OR Bifidobacteria OR *Bifidobacterium* OR *Candida robusta* OR *Clostridium butyricum* OR *Clostridium* OR *Fusobacterium* OR *Enterococcus* OR *Escherichia coli* OR *Eubacterium* OR Lactic acid bacteria OR *Lactobacill* OR *Lactobacillus* OR *Lactobacillus acidophilus* OR *Lactobacillus amylovorus* OR *Lactococcus* OR *Leuconostoc* OR Natto Bacteria OR *Peptostreptococcus* OR Prebiotics OR *Peptococcus* OR *S. cerevisiae* OR *S. cerevisiae* OR *Saccharomyces* OR *Saccharomyces capensis* OR *Saccharomyces cerevisiae* OR *Saccharomyces italicus* OR *Saccharomyces oviformis* OR *Saccharomyces u*var*um* var. *melibiosus* OR *Streptococcus* OR *Streptococcus thermophiles* OR Synbiotics OR Yeast OR Yogurt) AND (Quadruple OR Bismuth), without language, specific strains, or other restrictions. To further minimize the risk of missing relevant studies, we also manually searched the reference lists of relevant reviews in this field.

### 2.2 Inclusion and exclusion criteria

Inclusion criteria were as follows: (i) Participants: Patients with *H. pylori* infection confirmed by at least one validated diagnostic method (urea breath test, rapid urease test, bacterial culture, histology, or stool antigen test). (ii) Control group: The bismuth quadruple therapy as the standard regimen. (iii) Intervention group: Probiotics combined with standard bismuth quadruple therapy. (iv) Outcomes: The primary outcome was the *H. pylori* eradication rate. The secondary outcome was the adverse event rate. (v) Study design: RCTs.

Exclusion criteria were as follows: (i) Duplicate publications or retracted articles; (ii) Publications with unavailable full text or incomplete data; and (iii) Publications with obvious statistical errors.

### 2.3 Study selection

The study selection was a two-stage process performed by two independent reviewers. First, they screened titles and abstracts of studies retrieved from multiple databases against the predefined inclusion and exclusion criteria. Studies clearly irrelevant or not meeting the criteria were excluded. Disagreements were resolved through discussion or consultation with a third reviewer. Then, full-text articles of potentially eligible studies were retrieved and evaluated in depth. A PRISMA flow diagram was used to transparently present the selection process, ensuring a rigorous and objective selection of relevant studies for the review.

### 2.4 Data extraction

Two investigators independently performed data extraction using pre-designed standardized forms. The variables extracted from the original publications included the first author, publication year, country, sample size, gender distribution, mean patient age, treatment protocols for both intervention and control groups, probiotic strain, and treatment duration. The data from the included studies were independently extracted by two reviewers, and any disagreements were resolved by consensus.

### 2.5 Risk of bias assessment

The methodological quality of the included RCTs was independently assessed by two reviewers using the Cochrane Risk of Bias Assessment Tool (RoB 2.0). The RoB 2.0 tool evaluated five key domains: (i) bias arising from the randomization process; (ii) bias arising from deviations from the intended interventions; (iii) bias arising from missing outcome data; (iv) bias in the measurement of outcomes; and (v) bias in the selection of the reported result. A set of pre-specified signaling questions within each domain guided the reviewers to make judgments, classifying the risk of bias as “low,” “some concerns,” or “high.” Any discrepancies between the two reviewers were resolved through consensus discussions, and if necessary, a third reviewer was consulted to make a final decision. The overall assessment of each study was determined based on the ratings across all domains.

### 2.6 Statistical analysis

Meta-analyses were conducted by using Review Manager (version 5.3). Dichotomous outcomes, including *H. pylori* eradication rate and the adverse event rate, were synthesized as relative risks with corresponding 95 percent confidence intervals using the Mantel–Haenszel method. Statistical heterogeneity was quantified using both I^2^ and τ^2^. Given the expected clinical heterogeneity across the included trials, particularly differences in patient populations, probiotic strains and regimens, and variations in bismuth-containing quadruple therapy protocols, a random effects model was used as the primary analytical approach regardless of the I^2^ statistic. Additionally, for outcomes that reached statistical significance, absolute risk reduction (ARR) and number needed to treat (NNT) were calculated to enhance clinical interpretability.

To address potential sources of heterogeneity, prespecified subgroup analyses were conducted according to age group, probiotic formulation, timing of probiotic administration, treatment duration, and geographical region. Additionally, because included trials used different delta over baseline thresholds in the ^13^C urea breath test, ranging from three to five per mille, sensitivity analyses stratified by diagnostic cutoff were performed to evaluate the robustness of eradication outcomes and minimize diagnostic misclassification. Moreover, potential bias was further examined based on the results of the RoB 2 assessment. When a study was judged to have a high risk of bias or raised concerns in key domains, additional sensitivity analyses were conducted in which these studies were excluded, and the meta-analysis was repeated. This approach allowed evaluation of whether potential bias materially influenced the overall findings.

Assessment of publication bias followed established recommendations. When more than ten studies were available for a given outcome, funnel plots were generated by plotting effect estimates against their standard errors to visually assess asymmetry. In cases with ten or fewer studies, formal statistical tests were not performed due to limited power. Instead, a qualitative evaluation of potential publication bias was conducted, considering factors such as study size, the direction of effect, and consistency across trials.

### 2.7 Certainty of evidence

All assessments were conducted using GRADE pro GDT (version 3.6.1), with evidence profiles generated to support clinical decision-making. The certainty of evidence for meta-analysis outcomes was systematically evaluated using the Grading of Recommendations Assessment, Development, and Evaluation (GRADE) framework. RCTs were initially classified as high-quality evidence, with subsequent downgrading based on five predefined criteria: risk of bias, inconsistency, indirectness, imprecision, and others. Evidence quality was ultimately categorized into four levels: high, moderate, low, or very low.

## 3 Results

### 3.1 Literature search

**Figure 1:**
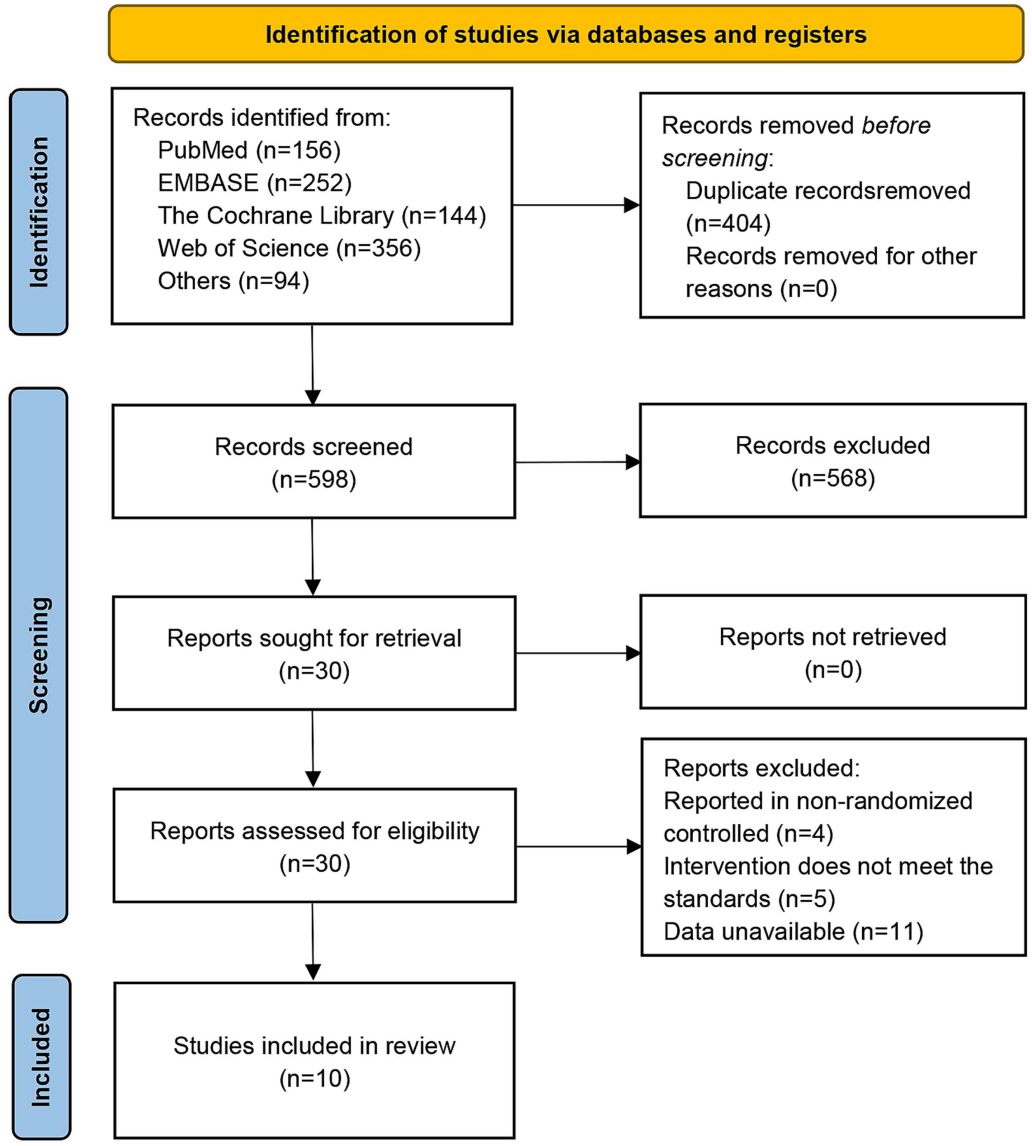
Flow diagram of the study selection process according to PRISMA guidelines.

A total of 1,002 studies were identified through screening. Specifically, 156 studies were retrieved from PubMed, 252 from Embase, 144 from the Cochrane Library, 356 from Web of Science, 84 from ClinicalTrials.gov, and 10 from reference lists. Among these studies, 404 duplicate studies were excluded by de-duplication, 568 studies were eliminated after reading the titles and abstracts, and 20 studies were excluded following full-text review. Finally, 10 studies were included, as shown in Figure 1.

### 3.2 Basic characteristics of included studies

The included studies were published between 2006 and 2024, all in full-text format. One study was conducted in Turkey (Bolat et al., 2022), while the remaining nine were carried out in China (Cai and Si, 2023; Chen et al., 2018; He et al., 2022; He et al., 2023; Jiang and Zhu, 2018; Sheu et al., 2006; Wang et al., 2024; Zhao et al., 2021; Zhou et al., 2024). The total sample size was 1,630 cases, including 798 cases in the experimental group and 832 cases in the control group. The average age of the participants ranged from 7.4 to 65.9 years, and the proportion of female participants was between 44.2 and 64.7%. Only one study had a treatment course of 1 week, and the treatment courses of the other studies were all 2 weeks. The basic characteristics are detailed in Table 1.

**Table 1:** Basic characteristics of included studies.

Study	Country	Sample (E/C)	Female (%)	Age (years)	Intervention	Comparison	Strain	Quadruple therapy duration (weeks)	Probiotic treatment duration (weeks)
Bolat et al. (2022)	Turkish	56/80	64.7	14.1	Lansoprazole 1 mg/kg/day, max 30 mg, tetracycline 50 mg/kg/day in 4 doses, max 4 × 500 mg, metronidazole 20 mg/kg/day, max 2 × 500 mg, and bismuth subsalicylate 262–524 mg qid; plus *B. lactis* B94 5 × 10^9^ CFU/d	Lansoprazole 1 mg/kg/day, max 30 mg, tetracycline 50 mg/kg/day in 4 doses, max 4 × 500 mg, metronidazole 20 mg/kg/day, max 2 × 500 mg, and bismuth subsalicylate 262–524 mg qid	*B. lactis* B94	2	2
Cai and Si (2023)	China	100/100	45.5	45.3	Esomeprazole magnesium enteric-coated tablets 20 mg bid, amoxicillin capsules 0.5 g bid, clarithromycin tablets 0.5 g bid, and colloidal pectin bismuth capsules 100 mg bid, plus *L. acidophilus* tablets 1 g bid	Esomeprazole magnesium enteric-coated tablets 20 mg bid, amoxicillin capsules 0.5 g bid, clarithromycin tablets 0.5 g bid, and colloidal pectin bismuth capsules 100 mg bid	*L. acidophilus*	2	2
Chen et al. (2018)	China	35/35	64.3	43.5	Pantoprazole 40 mg bid, amoxicillin 1,000 mg bid, furazolidone 100 mg bid, and colloidal bismuth pectin 0.4 g bid, plus *C. butyricum* 40 mg tid	Pantoprazole 40 mg bid, amoxicillin 1,000 mg bid, furazolidone 100 mg bid, and colloidal bismuth pectin 0.4 g bid	*Clostridium butyricum*	2	2
He et al. (2022)	China	90/90	50.0	65.9	Pantoprazole sodium enteric tablets 40 mg bid, colloidal bismuth pectin capsules 200 mg bid, amoxicillin capsules 1,000 mg bid, and clarithromycin sustained-release tablets 500 mg bid, plus *S. boulardii* powder 500 mg bid	Pantoprazole sodium enteric tablets 40 mg bid, colloidal bismuth pectin capsules 200 mg bid, amoxicillin capsules 1,000 mg bid, and clarithromycin sustained-release tablets 500 mg bid	*S. boulardii*	2	2
He et al. (2023)	China	86/86	45.9	41.6	Colloidal bismuth pectin 200 mg t.i.d., antofloxacin 200 mg q.i.d., esomeprazole 20 mg b.i.d., and amoxicillin 1,000 mg b.i.d., plus *S. boulardii* 500 mg b.i.d.	Colloidal bismuth pectin 200 mg t.i.d., antofloxacin 200 mg q.i.d., esomeprazole 20 mg b.i.d., and amoxicillin 1,000 mg b.i.d.	*S. boulardii*	2	2
Jiang and Zhu (2018)	China	111/111	44.1	35.0	Colloidal bismuth pectin 0.2 g bid, lansoprazole 15 mg bid, amoxicillin 1.0 g bid, and clarithromycin 0.5 g bid, plus live combined bifidobacterium 2 capsules tid.	Colloidal bismuth pectin 0.2 g bid, lansoprazole 15 mg bid, amoxicillin 1.0 g bid, and clarithromycin 0.5 g bid.	*Bifidobacterium*	2	2
Sheu et al. (2006)	China	69/69	44.2	47.7	Amoxicillin 1 g b.i.d, metronidazole 500 mg b.i.d, omeprazole 20 mg b.i.d, and bismuth subcitrate 120 mg t.i.d, plus *L. acidobacillus*– and *Bifidobacterium*-containing yogurt 200 mL b.i.d	Amoxicillin 1 g b.i.d, metronidazole 500 mg b.i.d, omeprazole 20 mg b.i.d, and bismuth subcitrate 120 mg t.i.d	*L. acidophilus* La5, *B. bifidum* Bb12, *Lactobacillus bulgaricus*, and *Streptococcus thermophilus*	1	4
Wang et al. (2024)	China	20/20	60.0	39.3	Rabeprazole 10 mg b.i.d, minocycline 100 mg b.i.d, amoxicillin 1,000 mg b.i.d, and bismuth potassium citrate 220 mg b.i.d, plus synbiotics 1 g/d	Rabeprazole 10 mg b.i.d, minocycline 100 mg b.i.d, amoxicillin 1,000 mg b.i.d, and bismuth potassium citrate 220 mg b.i.d	*Bifidobacterium* spp. and *Lactobacillus* spp.	2	6
Zhao et al. (2021)	China	169/179	47.4	46.0	Esomeprazole 20 mg bid, amoxicillin 1.0 g bid, clarithromycin 500 mg bid, and Bismuth potassium citrate 600 mg bid, plus *S. boulardii* sachets 500 mg bid	Esomeprazole 20 mg bid, amoxicillin 1.0 g bid, clarithromycin 500 mg bid, and Bismuth potassium citrate 600 mg bid	*S. boulardii*	2	2
Zhou et al. (2024)	China	62/62	47.6	7.4	Bismuth Potassium Citrate 1.2 g bid, Omeprazole 20 mg bid, Clarithromycin 250 mg bid, and Amoxicillin 1 g bid, plus Bifidobacterium quadruple viable tablets 1–1.5 g bid/tid, age-dependent.	Bismuth Potassium Citrate 1.2 g bid, Omeprazole 20 mg bid, Clarithromycin 250 mg bid, and Amoxicillin 1 g bid	*B. infantis*, *L. acidophilus*, *E. faecalis*, and *B. cereus*.	2	2

E, experimental group; C, control group.

### 3.3 Risk of bias assessment

**Figure 2:**
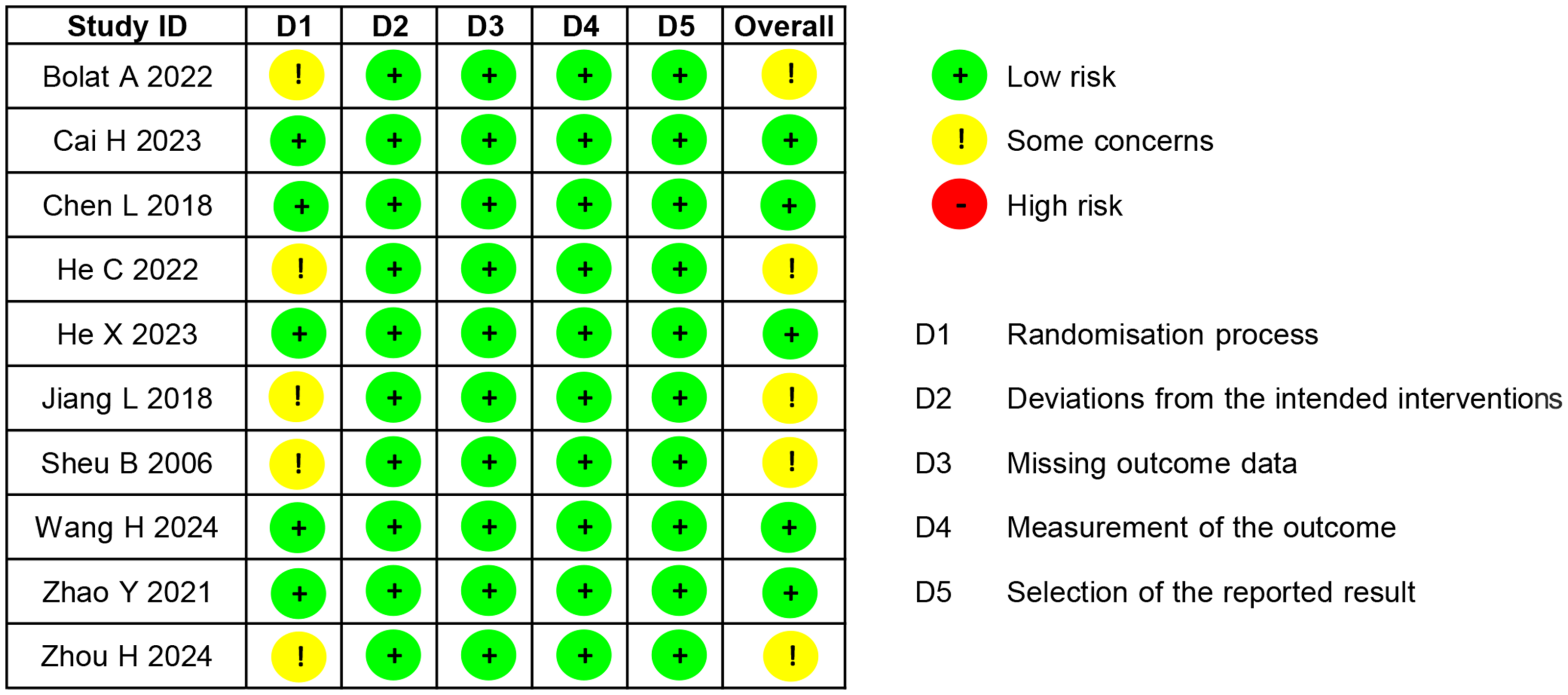
Summary of risk of bias for the included studies.

The risk of bias for the 10 included RCTs was assessed using the Cochrane RoB 2.0 tool. The overall and domain-specific judgments for each study are detailed in Figure 2. A summary of the judgments across all studies and the specific criteria applied is described below:

Bias arising from the randomization process (D1): Five studies were judged as “low risk” due to adequate descriptions of both random sequence generation (e.g., computer-generated random numbers) and allocation concealment (e.g., sealed opaque envelopes). However, five studies (Bolat et al., 2022; He et al., 2023; Jiang and Zhu, 2018; Sheu et al., 2006; Zhou et al., 2024) were rated as having “some concerns.” This judgment was made because, although these studies mentioned randomization, the method of allocation concealment was either not clearly described or its implementation could not be verified, introducing uncertainty about whether the allocation sequence was concealed before and during enrollment.

Bias due to deviations from the intended interventions (D2): All studies were judged to be at “low risk” because there were no deviations from the intended interventions caused by the trial environment.

Bias due to missing outcome data (D3): All studies were judged to be at “low risk.” The primary basis for this judgment is that the outcome data of each group were nearly complete, with a low missing rate for the primary outcomes. In addition, even if there was a small amount of missing data, the studies documented the reasons, and these reasons were unrelated to the intervention measures or outcomes.

Bias in measurement of the outcome (D4): All studies were rated “low risk.” This is because the primary outcome measures of the studies were confirmed by single or multiple objective laboratory methods (e.g., Urea Breath Test) and were not subject to subjective interpretation. There was no difference in the confirmation methods of outcomes between the two groups.

Bias in selection of the reported result (D5): All studies were classified as “low risk.” The reason is that the outcome indicators analyzed in the studies were consistent with the pre-specified plans. No multiple outcome measurements were conducted, and no multiple analysis methods were adopted.

In summary, although half of the included studies (*n* = 5) were judged to be at low overall risk of bias, the remaining studies (*n* = 5) were assessed as having some concerns, primarily related to issues in the randomization process, as detailed above.

### 3.4 Meta-analysis

The meta-analysis was conducted to evaluate the *H. pylori* eradication rate, adverse event rate, and other adverse events in the probiotics combined with the BQT group compared to the BQT group. The summary of findings is presented in Table 2.

**Table 2:** Summary of findings.

Outcome	I^2^/%	τ^2^	RR (95% CI)	*p*-value	ARR/%	NNT	Certainty
*H. pylori* eradication rate	34	0	1.06 (1.01, 1.11)	0.009	7.5	13	Low
Adverse event rate	54	0.11	0.58 (0.42, 0.80)	0.001	11.1	9	Very low
Nausea	0	0	0.73 (0.47, 1.14)	0.16	-	-	Very low
Vomiting	0	0	0.64 (0.40, 1.01)	0.05	-	-	Very low
Anorexia	0	0	0.66 (0.33, 1.30)	0.23	-	-	Very low
Heartburn	29	0.02	1.14 (0.85, 1.52)	0.39	-	-	Very low
Belching	0	0	0.82 (0.45, 1.49)	0.51	-	-	Very low
Taste disturbance	0	0	0.67 (0.44, 1.04)	0.08	-	-	Very low
Abdominal pain	12	0.02	0.83 (0.58, 1.18)	0.29	-	-	Very low
Abdominal bloating	60	0.49	0.65 (0.28, 1.51)	0.32	-	-	Very low
Diarrhea	38	0.13	0.48 (0.32, 0.73)	0.0007	0.7	142	Low
Constipation	0	0	0.53 (0.29, 0.97)	0.04	2.5	40	Low

RR, risk ratio; CI, confidence interval; *H. pylori*, *Helicobacter pylori*; ARR, absolute risk reduction; NNT, number needed to treat.

#### 3.4.1 *H. pylori* eradication rate

**Figure 3:**
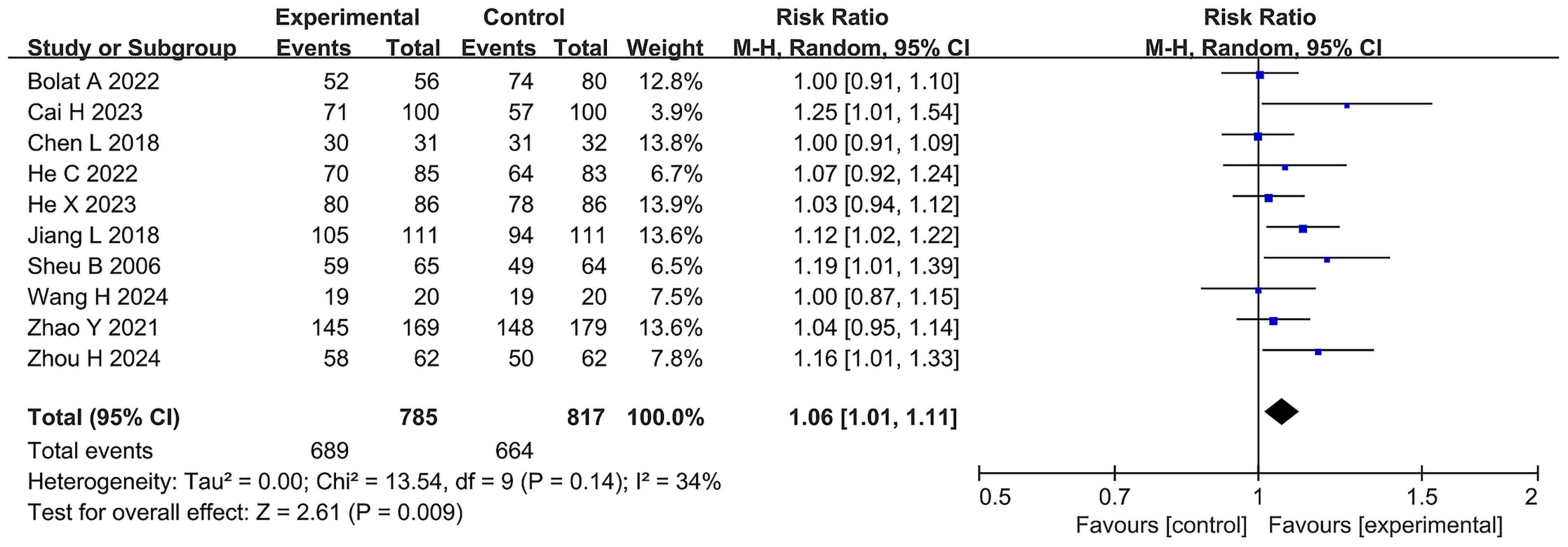
Forest plots of the meta-analysis on the *H. pylori* eradication rate.

A total of 1,602 patients from 10 RCTs were reported to have data on *H. pylori* eradication rate. The heterogeneity among the studies was moderate (I^2^ = 34%, τ*^2^* = 0). Meta-analysis showed that the *H. pylori* eradication rate was 81.27% in the BQT group and 88.78% in the group receiving probiotics combined with BQT therapy. Compared with the BQT group, the *H. pylori* eradication rate in the probiotics combined with the BQT group was significantly higher (RR 1.06, 95% CI 1.01–1.11, *p* = 0.009, ARR = 7.5%, NTT ≈ 13), as shown in Figure 3.

#### 3.4.2 Adverse event rate

**Figure 4:**
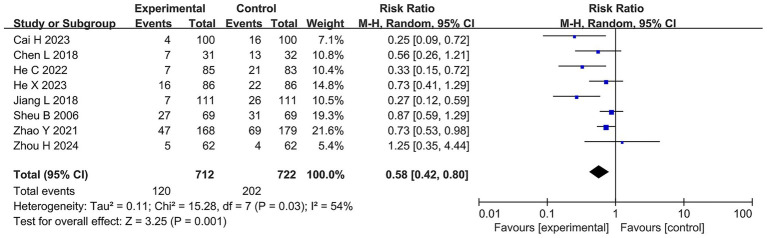
Forest plots of the meta-analysis on the adverse event rate.

Among all the studies, 8 RCTs reported the adverse event rate, with a total of 1,434 patients included. The heterogeneity test indicated high heterogeneity among the studies (I^2^ = 54%, τ*^2^* = 0.11). The results of the meta-analysis showed that the adverse event rate was 27.98% in the BQT group and 16.85% in the probiotics combined with the BQT group. Compared with the BQT group, the adverse event rate in the probiotics combined with the BQT group was significantly lower (RR 0.58, 95% CI 0.42–0.80, *p* = 0.001, ARR = 11.1%, NTT ≈ 9), as shown in Figure 4.

#### 3.4.3 Other adverse events

**Figure 5:**
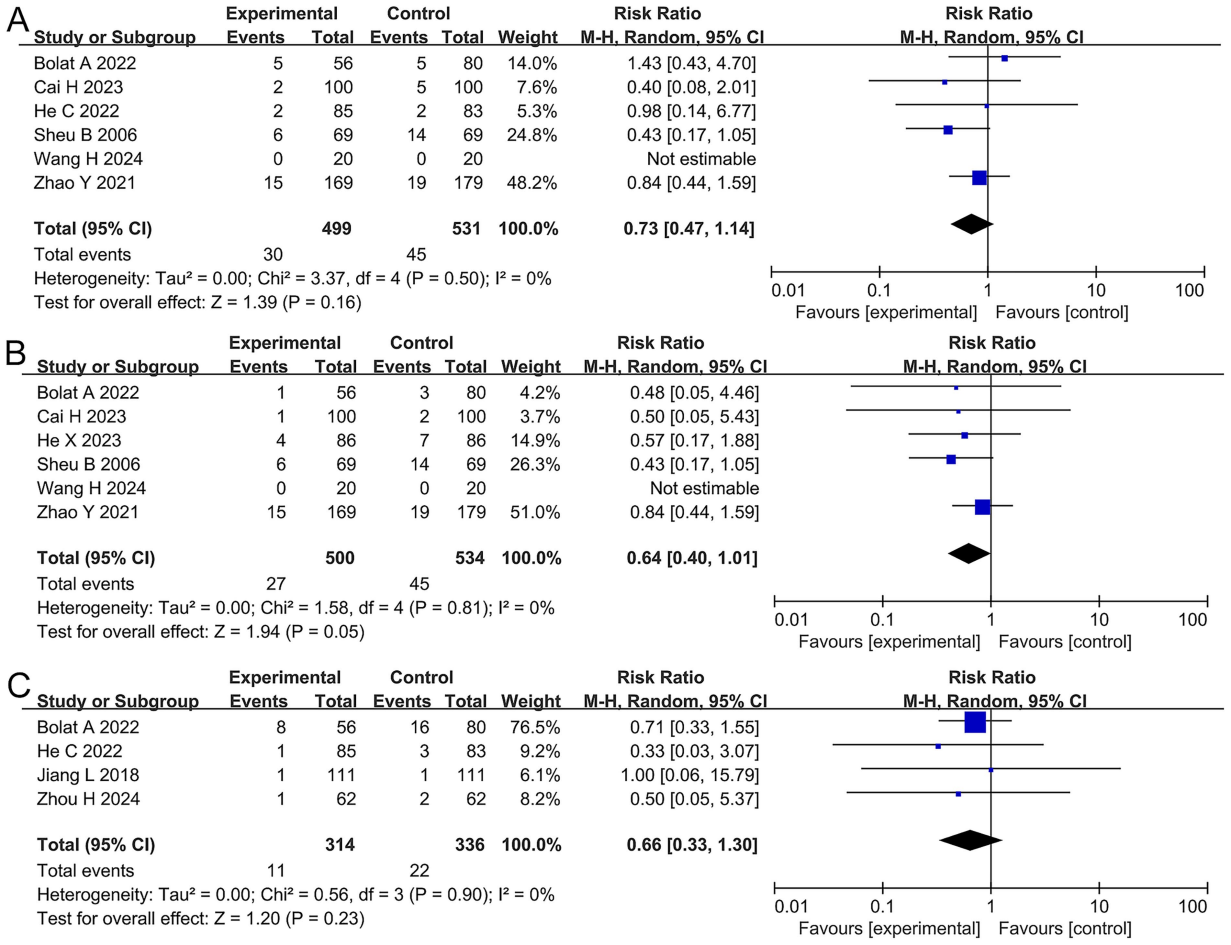
Forest plots of the meta-analysis on nausea, vomiting, and anorexia **(A–C)**.

Nausea, vomiting, and anorexia: The meta-analysis showed that there was no significant difference in the incidence of nausea (RR 0.73, 95% CI 0.47–1.14, *p* = 0.16), vomiting (RR 0.64, 95% CI 0.40–0.99, *p* = 0.045), or anorexia (RR 0.65, 95% CI 0.33–1.27, *p* = 0.20) between the two groups, as shown in Figure 5.

**Figure 6:**
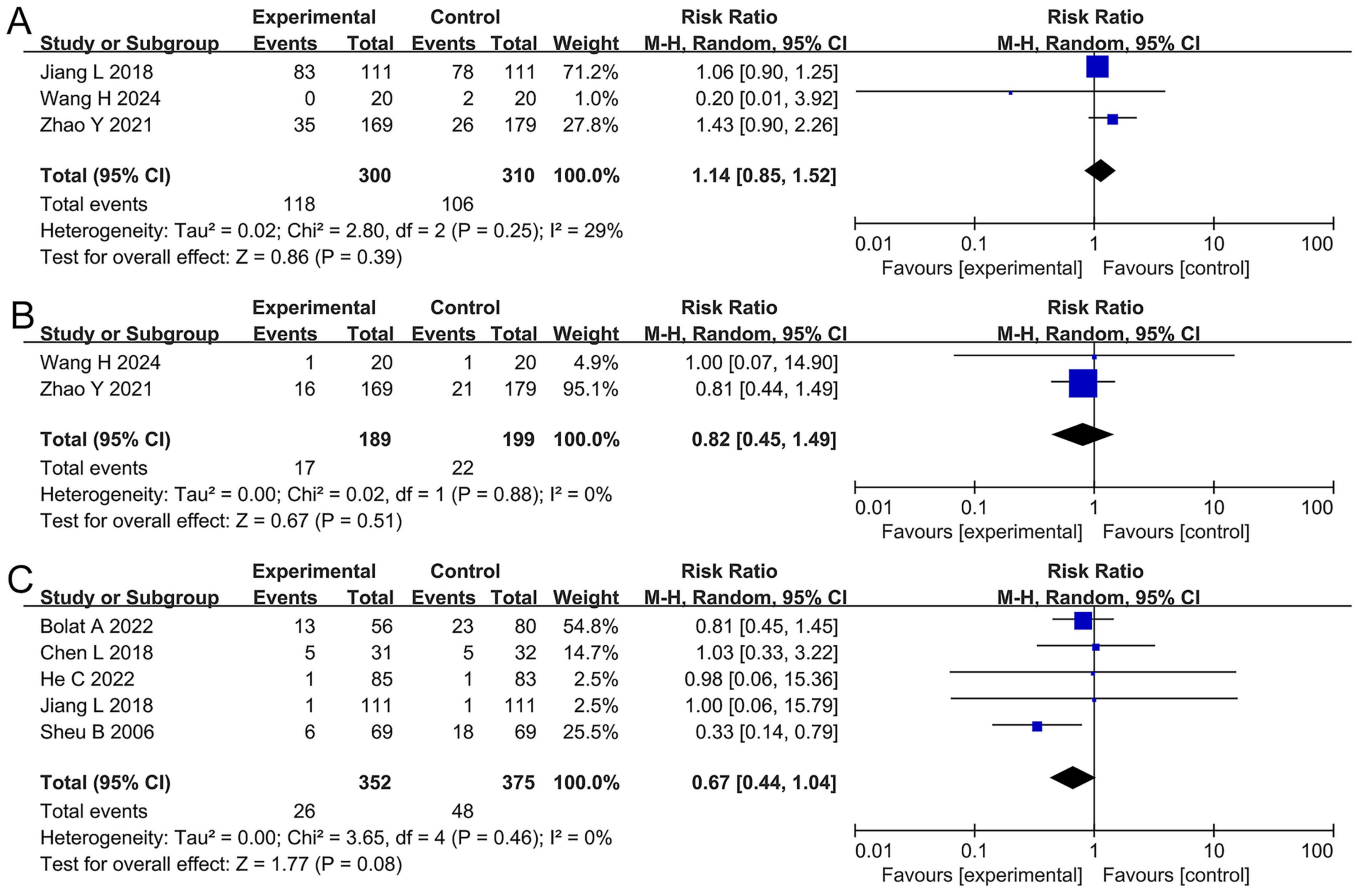
Forest plots of the meta-analysis on heartburn, belching, and taste disturbance. **(A)** Heartburn, **(B)** belching, and **(C)** taste disturbance.

Heartburn, belching, and taste disturbance: The meta-analysis indicated that there was no significant difference in the incidence of heartburn (RR 1.14, 95% CI 0.85–1.52, *p* = 0.39), belching (RR 0.82, 95% CI 0.45–1.49, *p* = 0.51), or taste disturbance (RR 0.67, 95% CI 0.44–1.04, *p* = 0.08) between the two groups, as shown in Figure 6.

**Figure 7:**
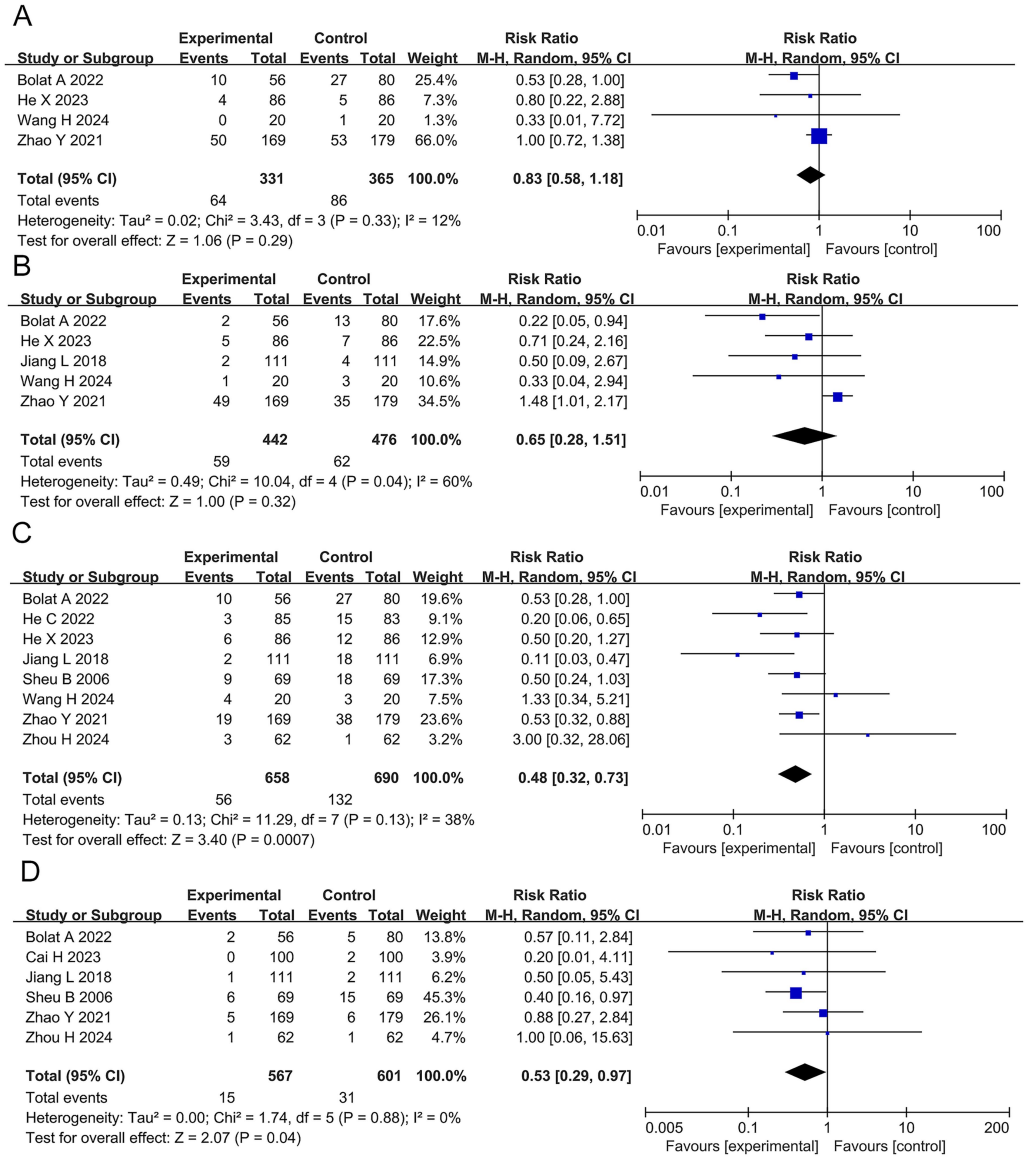
Forest plots of the meta-analysis on abdominal pain, abdominal bloating, diarrhea, and constipation **(A–D)**.

Abdominal pain, abdominal bloating, diarrhea, and constipation: The meta-analysis showed that, compared with the BQT group, the probiotics combined with BQT group significantly reduced the incidence of diarrhea (RR 0.48, 95% CI 0.32–0.73, *p* = 0.0007, ARR = 0.7%, NNT ≈ 142) and constipation (RR 0.53, 95% CI 0.29–0.94, *p* = 0.04, ARR = 2.5%, NNT ≈ 40). However, it had no significant effect on the incidence of abdominal pain (RR 0.83, 95% CI 0.58–1.18, *p* = 0.29) or abdominal bloating (RR 0.65, 95% CI 0.28–1.51, *p* = 0.32), as shown in Figure 7.

### 3.5 Subgroup analysis

Pre-specified subgroup analyses were conducted to explore the potential influence of clinical heterogeneity, including geographical region, populations, probiotic preparation, and BQT duration. Details are presented in Table 3. Since all included studies administered probiotics concurrently with BQT, subgroup analysis based on the timing of probiotic administration was not conducted.

**Table 3:** Subgroup analyses of clinical heterogeneity sources.

Outcome	Subject	Subgroup	Number of studies	I^2^/%	τ^2^	RR (95% CI)	*P*-value
*H. pylori* eradication rate	Geographical region	Turkish	1	0	0	1.00 (0.91, 1.10)	0.94
		China	9	33	0	1.07 (1.02, 1.12)	0.006
	Populations	Children	2	68	0.01	1.07 (0.92, 1.24)	0.37
		Adults	8	33	0	1.06 (1.01, 1.12)	0.02
	Probiotic preparation	Single-strain	7	26	0	1.07 (1.02, 1.12)	0.004
		Multi-strain	3	46	0	1.14 (1.05, 1.25)	0.004
	BQT duration	7 days	1	0	0	1.19 (1.01, 1.39)	0.03
		14 days	9	27	0	1.08 (1.03, 1.12)	0.0008
Adverse event rate	Populations	Children	1	0	0	1.25 (0.35, 4.44)	0.73
		Adults	6	58	0.11	0.55 (0.39, 0.78)	0.0007
	Probiotic preparation	Single-strain	6	55	0.13	0.49 (0.33, 0.73)	0.0004
		Multi-strain	2	0	0	0.90 (0.62, 1.31)	0.58
	BQT duration	7 days	1	0	0	0.87 (0.59, 1.29)	0.49
		14 days	7	52	0.12	0.52 (0.36, 0.76)	0.0008
Heartburn	Probiotic preparation	Single-strain	2	43	0.02	1.15 (0.86, 1.54)	0.33
		Multi-strain	1	0	0	0.20 (0.01, 3.92)	0.29
Abdominal bloating	Geographical region	Turkish	1	0	0	0.22 (0.05, 0.94)	0.04
		China	4	31	0.15	0.98 (0.52, 1.86)	0.96
	Populations	Children	1	0	0	0.22 (0.05, 0.94)	0.04
		Adults	4	31	0.15	0.98 (0.52, 1.86)	0.96
	Probiotic preparation	Single-strain	4	66	0.52	0.70 (0.29, 1.73)	0.44
		Multi-strain	1	0	0	0.33 (0.04, 2.94)	0.32
Diarrhea	Geographical region	Turkish	1	0	0	0.53 (0.28, 1.00)	0.05
		China	7	47	0.21	0.47 (0.28, 0.80)	0.005
	Populations	Children	2	54	0.82	0.90 (0.19, 4.33)	0.89
		Adults	6	43	0.16	0.43 (0.26, 0.72)	0.001
	Probiotic preparation	Single-strain	5	38	0.11	0.40 (0.25, 0.65)	0.0002
		Multi-strain	3	40	0.30	0.88 (0.34, 2.30)	0.80
	BQT duration	7 days	1	0	0	0.50 (0.24, 1.03)	0.06
		14 days	7	47	0.20	0.48 (0.29, 0.80)	0.005

RR, risk ratio; CI, confidence interval.

#### 3.5.1 *H. pylori* eradication rate

For geographical region, the subgroup analysis showed that probiotics significantly improved the *H. pylori* eradication rate among participants in China (RR 1.07, 95% CI 1.02–1.12, *p* = 0.006, I^2^ = 33%), whereas no significant effect was observed among participants in Turkey (RR 1.00, 95% CI 0.91–1.10, *p* = 0.94, I^2^ = 0%). For populations, the subgroup analysis indicated that probiotics significantly increased the *H. pylori* eradication rate in adults (RR 1.06, 95% CI 1.01–1.12, *p* = 0.02, I^2^ = 33%), while no significant effect was detected in children (RR 1.07, 95% CI 0.92–1.24, *p* = 0.37, I^2^ = 68%). For probiotic preparation, the subgroup analysis demonstrated that both single-strain (RR 1.07, 95% CI 1.02–1.12, *p* = 0.004, I^2^ = 26%) and multi-strain (RR 1.14, 95% CI 1.05–1.25, *p* = 0.004, I^2^ = 46%) formulations significantly improved the *H. pylori* eradication rate. For BQT duration, the subgroup analysis revealed that probiotics significantly enhanced the *H. pylori* eradication rate when combined with either 7-day (RR 1.19, 95% CI 1.01–1.39, *p* = 0.03, I^2^ = 0%) or 14-day BQT regimens (RR 1.08, 95% CI 1.03–1.12, *p* = 0.0008, I^2^ = 27%). In summary, these results indicate that the heterogeneity of the *H. pylori* eradication rate is not associated with geographical region, populations, probiotic preparation, or BQT duration.

#### 3.5.2 Adverse event rate

For populations, the subgroup analysis indicated that probiotics significantly reduced the adverse event rate in adults (RR 0.55, 95% CI 0.39–0.78, *p* = 0.0007, I^2^ = 58%), while no significant effect was observed in children (RR 1.25, 95% CI 0.35–4.44, *p* = 0.73, I^2^ = 0%). For probiotic preparation, the subgroup analysis revealed that single-strain probiotics significantly decreased the adverse event rate (RR 0.49, 95% CI 0.33–0.73, *p* = 0.0004, I^2^ = 55%), whereas multi-strain probiotics showed no significant effect (RR 0.90, 95% CI 0.62–1.31, *p* = 0.58, I^2^ = 0%). For BQT duration, the subgroup analysis demonstrated that probiotics significantly reduced the adverse event rate when combined with 14-day BQT regimens (RR 0.52, 95% CI 0.36–0.76, *p* = 0.0008, I^2^ = 52%), but not with 7-day regimens (RR 0.87, 95% CI 0.59–1.29, *p* = 0.49, I^2^ = 0%). In summary, these results indicate that the heterogeneity of the adverse event rate is not associated with populations, probiotic preparation, or BQT duration.

#### 3.5.3 Heartburn

For probiotic preparation, the subgroup analysis indicated that neither single-strain (RR 1.15, 95% CI 0.86–1.54, *p* = 0.33, I^2^ = 43%) nor multi-strain formulations (RR 0.20, 95% CI 0.01–3.92, *p* = 0.29, I^2^ = 0%) showed a significant effect on heartburn. It suggests that the heterogeneity of heartburn may be associated with the type of probiotic preparation.

#### 3.5.4 Abdominal bloating

For geographical region, subgroup analysis revealed that probiotics significantly reduced abdominal bloating in participants from Turkey (RR 0.22, 95% CI 0.05–0.94, *p* = 0.04, I^2^ = 0%), while no significant effect was observed among participants in China (RR 0.98, 95% CI 0.52–1.86, *p* = 0.96, I^2^ = 31%). For populations, the subgroup analysis demonstrated that probiotics significantly reduced the risk of abdominal bloating in children (RR 0.22, 95% CI 0.05–0.94, *p* = 0.04, I^2^ = 0%), while no significant effect was observed in adults (RR 0.98, 95% CI 0.52–1.86, *p* = 0.96, I^2^ = 31%). For probiotic preparation, the subgroup analysis indicated that neither single-strain (RR 0.70, 95% CI 0.29–1.73, *p* = 0.44, I^2^ = 66%) nor multi-strain formulations (RR 0.33, 95% CI 0.04–2.94, *p* = 0.32, I^2^ = 0%) significantly reduced the risk of abdominal bloating. In summary, these findings imply that the heterogeneity of abdominal bloating in response to probiotic treatment may be influenced by geographical location and population characteristics.

#### 3.5.5 Diarrhea

For geographical region, the subgroup analysis showed that probiotics significantly reduced the incidence of diarrhea among participants in China (RR 0.47, 95% CI 0.28–0.80, *p* = 0.005, I^2^ = 47%), while a marginal effect was observed among participants in Turkey (RR 0.53, 95% CI 0.28–1.00, *p* = 0.05, I^2^ = 0%). For populations, the subgroup analysis indicated that probiotics significantly decreased diarrhea in adults (RR 0.43, 95% CI 0.26–0.72, *p* = 0.001, I^2^ = 43%), whereas no significant effect was found in children (RR 0.90, 95% CI 0.19–4.33, *p* = 0.89, I^2^ = 54%). For probiotic preparation, the subgroup analysis demonstrated that single-strain formulations significantly reduced diarrhea (RR 0.40, 95% CI 0.25–0.65, *p* = 0.0002, I^2^ = 38%), while multi-strain formulations did not show a significant effect (RR 0.88, 95% CI 0.34–2.30, *p* = 0.80, I^2^ = 40%). For BQT duration, the subgroup analysis revealed that probiotics significantly lowered the incidence of diarrhea when combined with 14-day BQT regimens (RR 0.48, 95% CI 0.29–0.80, *p* = 0.005, I^2^ = 47%), while a trend was observed with 7-day BQT (RR 0.50, 95% CI 0.24–1.03, *p* = 0.06, I^2^ = 0%). In summary, these findings suggest that the heterogeneity in the effect of probiotics on diarrhea may be influenced by clinical factors such as geographical region, probiotic preparation, and BQT duration.

### 3.6 Sensitivity analysis based on methodological quality and diagnostic cutoff

We excluded studies with some concern in the randomization process to assess the impact of methodological heterogeneity, as shown in Table 4. After removing studies with unclear risk, sensitivity analyses showed that probiotics significantly improved the *H. pylori* eradication rate (RR 1.07, 95% CI 1.00–1.13, *p* = 0.04, I^2^ = 32%), and significantly reduced the overall adverse event rate (RR 0.64, 95% CI 0.46–0.88, *p* = 0.007, I^2^ = 24%). For specific adverse events, probiotics significantly reduced the rate of diarrhea (RR 0.57, 95% CI 0.37–0.88, *p* = 0.01, I^2^ = 0%), but had no significant effect on heartburn (RR 0.94, 95% CI 0.19–4.58, *p* = 0.94, I^2^ = 40%) or abdominal bloating (RR 1.07, 95% CI 0.53–2.14, *p* = 0.85, I^2^ = 36%). This analysis suggests that the heterogeneity observed in the adverse event rate, heartburn, abdominal bloating, and diarrhea may be related to the inclusion of studies with potential bias.

**Table 4:** Sensitivity analyses based on methodological quality and diagnostic cutoff.

Subject	Outcome	I^2^/%	τ^2^	RR (95% CI)	*P*-value
Methodological quality	*H. pylori* eradication rate	32	0	1.07 (1.00, 1.13)	0.04
	Adverse event rate	24	0.03	0.64 (0.46, 0.88)	0.007
	Heartburn	40	0.77	0.94 (0.19, 4.58)	0.94
	Abdominal bloating	36	0.16	1.07 (0.53, 2.14)	0.85
	Diarrhea	0	0	0.57 (0.37, 0.88)	0.01
Diagnostic cutoff	*H. pylori* eradication rate	18	0	1.05 (1.01, 1.10)	0.01
	Adverse event rate	49	0.08	0.62 (0.45, 0.85)	0.003

RR, risk ratio; CI, confidence interval.

Additionally, Cai and Si, 2023 applied varying *Δ*-over-baseline cutoffs (3–5‰) in the ^13^C-urea breath test, which may have introduced misclassification bias in determining eradication. Therefore, we conducted a sensitivity analysis excluding studies with inconsistent diagnostic thresholds, as shown in Table 4. The results demonstrated that probiotics significantly improved the *H. pylori* eradication rate (RR 1.05, 95% CI 1.01–1.10, *p* = 0.01, I^2^ = 18%) and significantly reduced the adverse event rate (RR 0.62, 95% CI 0.45–0.85, *p* = 0.003, I^2^ = 49%). These findings suggest that the heterogeneity observed in the *H. pylori* eradication rate and adverse event rate may be related to differences in diagnostic cutoffs. However, because the testing protocols varied substantially across studies, further subgroup or sensitivity analyses based on diagnostic methods could not be performed.

### 3.7 Sensitivity analysis based on the leave-one-out method

To evaluate the robustness of the pooled effect size and the impact of sources of heterogeneity, this study employed the leave-one-out analysis method to conduct sensitivity analysis on outcomes with existing heterogeneity (Table 5). The results are as follows:

**Table 5:** Leave-one-out sensitivity analyses of identified heterogeneous sources.

Outcome	Heterogeneity source	Sensitivity analysis results			Robustness
		I^2^/%	RR (95% CI)	*P*-value	
*H. pylori* eradication rate	Cai and Si (2023)	18	1.05 (1.01, 1.10)	0.01	Robust
Adverse event rate	Jiang and Zhu (2018)	39	0.65 (0.49, 0.87)	0.004	Robust
Heartburn	Zhao et al. (2021)	21	0.89 (0.31, 2.53)	0.82	Robust
Abdominal bloating	Zhao et al. (2021)	0	0.45 (0.22, 0.94)	0.03	Not robust
Diarrhea	Jiang and Zhu (2018)	11	0.53 (0.38, 0.74)	0.0002	Robust

RR, risk ratio; CI, confidence interval.

#### 3.7.1 *H. pylori* eradication rate

The heterogeneity in the *H. pylori* eradication rate originated from the study by Cai and Si (2023). After excluding this study, the heterogeneity significantly decreased (I^2^ = 18%, τ*^2^* = 0), and the effect assessment remained statistically significant (RR 1.05, 95% CI 1.01–1.10, *p* = 0.01), which confirms the reliability of the main conclusion. Cai and Si (2023) adopted a Delta Over Baseline (DOB) threshold of ≥ 4.0 ± 0.4 for the ^13^C-urea breath test, whereas most studies used a fixed value (DOB ≥ 4.0). The inconsistency in the DOB judgment criteria may increase the risk of false negatives and lead to detection bias, resulting in misestimation of the eradication rate.

#### 3.7.2 Adverse event rate

After excluding the study by Jiang and Zhu (2018), the heterogeneity significantly decreased (I^2^ = 39%, τ*^2^* = 0.05), and the pooled effect size remained statistically significant (RR 0.65, 95% CI 0.49–0.87, *p* = 0.004), indicating that the results of the meta-analysis are robust. The heterogeneity may be related to the use of live combined *Bifidobacterium* as the bacterial strain: in the study, *Bifidobacterium* mainly improved diarrhea but had a weak effect on abdominal bloating and constipation, leading to a deviation of its effect size from that of other studies.

#### 3.7.3 Heartburn

After excluding the study by Zhao et al. (2021), the results showed high consistency (I^2^ = 21%, τ*^2^* = 0.31), and there were no substantial changes in either the direction of the effect or its statistical significance (RR 0.89, 95% CI 0.31–2.53, *p* = 0.82), which confirms the robustness of the meta-analysis results. In the study by Zhao et al.(Zhao et al., 2021), the heartburn relief rate was 81.4% in the intervention group and 76.5% in the control group, with an improvement magnitude significantly smaller than that in other studies. This suggests that different bacterial strains have varying effects on improving heartburn symptoms, which is one of the important factors contributing to heterogeneity. Additionally, *Saccharomyces boulardii* used in Zhao et al. (2021) primarily targeted the improvement of diarrhea-related adverse events; in the study, patients with severe diarrhea were additionally given montmorillonite powder. Montmorillonite powder can relieve heartburn while treating diarrhea, which may lead to misestimation of the symptom relief rate and introduce confounding bias, thereby causing heterogeneity.

#### 3.7.4 Abdominal bloating

After excluding the study by Zhao et al. (2021), the heterogeneity was eliminated (I^2^ = 0%, τ*^2^* = 0), and the originally non-significant effect was converted into a statistically significant protective effect (RR 0.45, 95% CI 0.22–0.94, *p* = 0.03). This significant change suggests that the pooled result for abdominal bloating is likely affected by heterogeneity dominated by a single study, and its reliability is insufficient to a certain extent. Therefore, the conclusion regarding the risk of abdominal bloating requires cautious interpretation. The pooled effect size of abdominal bloating showed high sensitivity to the study by Zhao et al. (2021), which may be the main source of heterogeneity. The reasons may be as follows: different probiotic strains have specificity, which may affect the effect of probiotics on improving abdominal bloating; in addition, in this study, patients with severe diarrhea were given additional montmorillonite powder, and this intervention may have interfered with the effect of probiotics on abdominal bloating, thereby causing the abdominal bloating result of this study to deviate from the true effect.

#### 3.7.5 Diarrhea

Sensitivity analysis after excluding the study by Jiang and Zhu (2018) showed minimal heterogeneity (I^2^ = 11%, τ*^2^* = 0.02) and an enhanced effect size (RR 0.53, 95% CI 0.38–0.74, *p* = 0.0002). This indicates that the meta-analysis results are robust and further confirms the conclusion that probiotics can significantly reduce the risk of diarrhea. The heterogeneity may be attributed to the following: the study used live combined *Bifidobacterium*, which had a significant effect on improving diarrhea, leading to a substantial reduction in the incidence of diarrhea in the probiotic group of this study and a prominent difference in diarrhea improvement effects compared with other studies. Meanwhile, the study used lansoprazole at a dose of 15 mg twice daily (bid), which was significantly lower than the conventional dose (30 mg bid). This insufficient dose may have failed to adequately inhibit gastric acid secretion, thereby affecting the effect of probiotics on diarrhea improvement and becoming a source of heterogeneity.

In summary, identified sources of heterogeneity include the following: for the *H. pylori* eradication rate, inconsistent ^13^C-urea breath test diagnostic thresholds contributed substantially; for the adverse event rate, heterogeneity was mainly associated with inclusion of studies with potential bias in randomization and with variation in probiotic strains, particularly the study using live combined *Bifidobacterium*; for heartburn, strain specificity and confounding co-interventions such as montmorillonite powder drove inconsistent effects; for abdominal bloating, geographic region and population characteristics were important contributors; and for diarrhea, heterogeneity was influenced by geographical region, probiotic formulation, BQT duration, and study-specific factors such as the use of live combined Bifidobacterium and atypical PPI dosing.

### 3.8 Publication bias

Because none of the outcomes included more than ten studies, funnel plots were not generated. Instead, a qualitative evaluation suggested that potential publication bias may be present across the included evidence. Several common factors likely contributed to this risk. First, many of the trials were small-scale, single-center studies, which increases the likelihood that statistically significant or favorable findings are more readily published than neutral or negative results. Second, research involving probiotic supplementation tends to exhibit a positive publication tendency, as studies reporting beneficial effects may be more likely to be submitted, accepted, or disseminated. Third, substantial variability in outcome measurement and reporting practices, particularly for adverse events, may allow selective emphasis on beneficial findings. Together, these factors suggest that the body of evidence may be influenced by publication bias, and the observed effect sizes should be interpreted with appropriate caution.

### 3.9 Certainty of evidence

According to the GRADE guidelines, the certainty of evidence was assessed as low for the *H. pylori* eradication rate, diarrhea, and constipation. Additionally, the certainty of evidence was assessed as very low for the adverse event rate, nausea, vomiting, anorexia, heartburn, belching, taste disturbance, abdominal pain, and abdominal bloating. Given the overall low to very low certainty of the evidence, the strength of the recommendations is accordingly weak, as shown in Table 6.

**Table 6:** Certainty of evidence.

Outcome	Risk of bias	Inconsistency	Indirectness	Imprecision	Others	RR (95% CI)	Certainty of evidence
*H. pylori* eradication rate	Serious	None	None	None	Publication bias	1.06 (1.01, 1.11)	Low
Adverse event rate	Serious	Serious	None	None	Publication bias	0.58 (0.42, 0.80)	Very low
Nausea	Serious	None	None	Serious	Publication bias	0.73 (0.47, 1.14)	Very low
Vomiting	Serious	None	None	Serious	Publication bias	0.64 (0.40, 1.01)	Very low
Anorexia	Serious	None	None	Serious	Publication bias	0.66 (0.33, 1.30)	Very low
Heartburn	Serious	None	None	Serious	Publication bias	1.14 (0.85, 1.52)	Very low
Belching	Serious	None	None	Serious	Publication bias	0.82 (0.45, 1.49)	Very low
Taste disturbance	Serious	None	None	Serious	Publication bias	0.67 (0.44, 1.04)	Very low
Abdominal pain	Serious	None	None	Serious	Publication bias	0.83 (0.58, 1.18)	Very low
Abdominal bloating	Serious	Serious	None	Serious	Publication bias	0.65 (0.28, 1.51)	Very low
Diarrhea	Serious	None	None	None	Publication bias	0.48 (0.32, 0.73)	Low
Constipation	Serious	None	None	None	Publication bias	0.53 (0.29, 0.97)	Low

RR, risk ratio; CI, confidence interval.

## 4 Discussion

As a currently recommended first-line treatment regimen, BQT can stably maintain the *H. pylori* eradication rate at approximately 90% (Yin et al., 2023). However, this therapy has issues such as a complex administration schedule and a relatively high adverse event rate. In addition, BQT may also significantly reduce the diversity of intestinal flora and promote an increase in the relative abundance of opportunistic pathogens such as Proteobacteria (e.g., *Escherichia coli*) (Zhao et al., 2024). At present, pharmacological intervention remains the main clinical strategy for managing gastrointestinal-related adverse events, but conventional treatments like antibiotics may further disrupt the balance of intestinal microecology. Our study demonstrates that adjuvant probiotics with BQT robustly improve the *H. pylori* eradication rate and significantly reduce the adverse event rate, diarrhea, and constipation. It is important to emphasize, however, that the certainty of the evidence supporting these findings is low to very low; therefore, our recommendation for this strategy remains conditional.

Both our meta-analysis and sensitivity analysis support that the combination of probiotics with BQT can improve the *H. pylori* eradication rate, which strengthens the clinical value of probiotics as an adjuvant therapy. A number of meta-analyses (Liang et al., 2023; Shi et al., 2019; Yao et al., 2023) have also confirmed the clear value of probiotics in *H. pylori* eradication treatment: the combined use of probiotics and BQT therapy can increase the overall eradication rate and improve patient tolerance by reducing the gastrointestinal irritation caused by BQT. In addition, the combined use of probiotics and antibiotics can enhance the inhibition rate and eradication rate of antibiotics against *H. pylori* (Emara et al., 2014; Lionetti et al., 2006). There is evidence that adding probiotics to the quadruple *H. pylori* eradication regimen may improve the eradication rate and reduce the occurrence of new symptoms related to the eradication regimen or the exacerbation of existing symptoms of any severity (Viazis et al., 2022). Furthermore, relevant studies have also confirmed that BQT therapy with added probiotics can achieve an excellent eradication rate and cause fewer side effects even in patients who failed previous eradication treatments (Zhu et al., 2018; Bai et al., 2022). These pieces of evidence indicate that probiotics can serve as an adjuvant strategy for BQT to promote the eradication of *H. pylori*.

The mechanisms by which probiotics improve the *H. pylori* eradication rate mainly involve two aspects: immunomodulation and non-immunomodulation. Probiotics can enhance the host’s immunity and activate mucosal protection mechanisms through multiple pathways (Yang et al., 2012; Plaza-Díaz et al., 2017) to control *H. pylori* infection. Not only do they modulate immune responses by elevating anti-inflammatory factors and reducing pro-inflammatory cytokine levels, thereby protecting the host from tissue damage and maintaining T-cell homeostasis (Zhao et al., 2021), but they also ameliorate local inflammation by inhibiting signaling pathways such as Smad7 and NF-κB (Yang et al., 2012). In addition, probiotics can regulate the number of macrophages and dendritic cells (Plaza-Díaz et al., 2017) to reduce inflammatory responses. For example, some lactobacilli can inhibit the inflammatory functions of lymphocyte subsets (Th1 and Th17) and their cytokines; at the same time, they stimulate dendritic cells to form anti-inflammatory pathways and promote B cells to produce IgA (Eslami et al., 2019), thereby strengthening the immune barrier and effectively promoting mucosal repair. Notably, the non-immunomodulatory mechanisms of probiotics are equally important. Endowed with strong adhesion ability, probiotics adhere to gastric epithelial cells, compete with *H. pylori*, and prevent *H. pylori* from colonizing the gastrointestinal tract (Li et al., 2022). They produce inhibitory metabolites such as lactic acid, short-chain fatty acids, hydrogen peroxide, and bacteriocins (He et al., 2023) to inhibit the urease activity of *H. pylori*; meanwhile, they release bacteriocins to disrupt the integrity of pathogen cell membranes and suppress pathogen growth. Additionally, probiotics can synergistically inhibit the expression of key virulence genes like VacA and flaA (Urrutia-Baca et al., 2017). They also maintain the integrity of the mucus layer by promoting mucin secretion, enhancing the intestinal mucosal barrier function (Wang et al., 2020), and protecting the intestinal epithelium from pathogen invasion. Therefore, the use of probiotics holds significant clinical value in controlling *H. pylori* infection and restoring the microbiota after *H. pylori* eradication.

In terms of safety assessment, our meta-analysis supports that probiotics reduce the adverse event rate as well as individual gastrointestinal adverse events such as diarrhea and constipation, which is consistent with previous reports. Previous studies have shown that *Lactobacillus rhamnosus* and *Lactobacillus acidophilus* work synergistically to improve defecation frequency and constipation symptoms (Wang et al., 2022). The underlying mechanism may be related to their production of short-chain fatty acids, which can inhibit the growth of pathogenic bacteria either directly or by lowering the intestinal pH value (Rinttilä and Apajalahti, 2013); *Bifidobacterium* improves constipation by regulating the relative abundance of bacteria related to gastrointestinal regulatory peptides (Jiang et al., 2022); *Lactobacillus reuteri* regulates the intestinal microbiota to alleviate inflammation and antibiotic-associated diarrhea (Lu et al., 2022); *Saccharomyces boulardii*, a probiotic widely used in the prevention or treatment of intestinal diseases, reduces the risk of antibiotic-associated diarrhea through its anti-inflammatory and antibacterial activities, while increasing the *H. pylori* eradication rate and reducing intestinal inflammatory responses (Chen et al., 2009); *Clostridium butyricum* alleviates gastrointestinal inflammation by producing butyric acid (Shin et al., 2023) and inhibits the proliferation of intestinal pathogenic microorganisms and harmful bacteria (Ma et al., 2022). Notably, these adverse events are generally considered to be associated with BQT and are common factors affecting treatment adherence. It has been reported that the gastrointestinal adverse events (such as diarrhea, abdominal bloating, and constipation) accompanying BQT treatment are partly caused by drug effects, such as antibiotic-associated diarrhea and PPI-induced intestinal microbiota disorders (Hojo et al., 2018; Motamedi et al., 2021). In contrast, probiotics can alleviate gastrointestinal adverse events caused by antibiotics and PPIs by restoring the balance of intestinal microbiota (Wang et al., 2022), including but not limited to diarrhea, constipation, and dyspepsia (Mohtasham et al., 2023; Marinelli et al., 2022; Márquez et al., 2021). Although limited by specific bacterial strains and treatment courses, there remains controversy regarding whether probiotics can reduce the risk of headache and abdominal bloating (Mohtasham et al., 2023; Márquez et al., 2021); their value in improving the risk of diarrhea and constipation has been consistently verified by similar clinical studies and meta-analyses (Liang et al., 2023; Yao et al., 2023; Zhao et al., 2025; Han et al., 2024). These pieces of evidence indicate that probiotics can alleviate gastrointestinal adverse events during BQT treatment, which helps improve patients’ treatment adherence. Currently, the World Gastroenterology Organization Global Guidelines on Probiotics and Prebiotics (2024) state that probiotics play a role in the treatment of various gastrointestinal diseases, and guidelines in many countries have incorporated probiotic preparations into adjuvant treatment strategies for multiple gastrointestinal diseases (Guarino et al., 2018). For example, the British Society of Gastroenterology Guidelines for the Management of Irritable Bowel Syndrome (IBS) (2021) (Vasant et al., 2021) suggests that probiotics may be an effective approach for treating the overall symptoms and abdominal pain of IBS; the Japanese Evidence-Based Clinical Practice Guidelines for IBS (2020) (Fukudo et al., 2021) clearly states that probiotics can be used for the treatment of Irritable Bowel Syndrome subtypes; the Evidence-Based Clinical Guidelines for Chronic Diarrhea (2023) (Ihara et al., 2024) points out that oral probiotics with intestinal microbiota-regulating and anti-diarrheal effects should be considered first for treatment; and it is recommended to supplement probiotics during the eradication regimen to reduce adverse events, improve adherence, and enhance eradication effectiveness (Shin et al., 2023).

Probiotics play a crucial role in alleviating gastrointestinal adverse events through multiple mechanisms. First, probiotics can regulate endocrine function via the “microbiota-gut-brain axis” (Uhlig et al., 2020), strengthen the intestinal barrier, and inhibit the adhesion of pathogenic bacteria to the intestinal wall, thereby improving gastrointestinal symptoms such as abdominal pain and constipation in patients (Xiang et al., 2022). Second, intestinal epithelial cells sense probiotic stimulation through specialized enteroendocrine cells, converting it into neuroendocrine signals (Uhlig et al., 2020) that are transmitted to the central nervous system via the vagus nerve to regulate the normal motility of the gastrointestinal tract. Third, the increase in probiotics exerts its effects by reducing the production of endotoxins, enhancing intestinal barrier function, and alleviating inflammation (Yuan et al., 2019). Fourth, probiotics can also regulate gastrointestinal immune responses and reduce drug-induced inflammatory reactions by inhibiting inflammation and oxidative damage (Mahdy et al., 2023). Finally, probiotic supplementation leads to an increase in the content of short-chain fatty acids (Huang et al., 2022). Short-chain fatty acids not only promote intestinal peristalsis and improve the balance of intestinal microbiota but also possess anti-inflammatory effects, which can alleviate intestinal adverse events (Sun et al., 2022). In summary, these studies provide an explanation for the mechanism by which probiotics reduce gastrointestinal adverse events and indirectly support the application of probiotics in *H. pylori* eradication.

Notably, the magnitude of benefit from probiotics exhibits clear strain specificity and formula dependence, indicating that not all probiotic preparations exert equivalent effects. Regarding improvement in eradication rates, although our subgroup analyses showed that both single-strain and multi-strain preparations confer significant advantages, a closer examination of individual strains reveals deeper distinctions. For example, a recently published meta-analysis confirmed that *Saccharomyces boulardii* is one of the few strains capable of significantly enhancing eradication rates (Li and Xie, 2025). Similarly, *Lactobacillus reuteri* also demonstrated remarkable eradication efficacy in a newly released 2025 meta-analysis (Elkoumi et al., 2025). In contrast, certain Lactobacillus strains used in some studies showed relatively limited ability to increase eradication rates when administered alone (Javanmard et al., 2018; Zaman et al., 2024). Strain-specific effects are equally evident in reducing adverse events. *S. boulardii* has been shown to broadly and significantly decrease multiple adverse events (Li and Xie, 2025), with particularly notable protective effects against gastrointestinal symptoms such as diarrhea. Similarly, some multi-strain formulations—such as those combining *Lactobacillus* and *Bifidobacterium* species—have demonstrated improved overall treatment tolerability through synergistic interactions among strains (Wang et al., 2023). Taking both eradication efficacy and safety enhancement into consideration, we recommend giving priority to strains supported by high-level evidence for improving eradication outcomes while simultaneously reducing adverse events, such as *S. boulardii*. This is consistent with the Maastricht VI/Florence 2022 consensus, which explicitly states that specific probiotics (notably *S. boulardii*) can mitigate treatment-related side effects (Malfertheiner et al., 2022).

Several limitations of this meta-analysis should be acknowledged. First, the number of included studies and the overall sample size were relatively small, which may reduce the statistical power and precision of the pooled estimates. Second, some original trials were at risk of selection bias and performance bias, potentially affecting the reliability of the findings. Third, nine out of the ten included randomized controlled trials were conducted in China, which substantially limits the external validity of the results. This geographic concentration raises concerns about extrapolation to regions with different epidemiological and therapeutic contexts. For example, the American College of Gastroenterology (ACG) Clinical Guideline on the Treatment of *Helicobacter pylori* Infection (Chey et al., 2024) recommends optimized bismuth quadruple therapy or vonoprazan-based regimens as first-line treatment, while the Maastricht VI/Florence 2022 (Malfertheiner et al., 2022) consensus also emphasizes regional antibiotic resistance patterns, particularly clarithromycin and amoxicillin resistance, when selecting empirical therapy. In contrast, most included studies used conventional bismuth quadruple therapy with amoxicillin, which may not reflect current practices in regions where vonoprazan-containing therapies predominate or where amoxicillin resistance is high. These differences in guideline-recommended regimens and antimicrobial resistance profiles may limit the generalizability of our findings outside East Asia. Finally, the optimal probiotic strain combination, timing of administration, and treatment duration remain unclear, resulting in the absence of standardized recommendations for clinical use. To address these limitations, future studies should include large, multi-center randomized controlled trials across diverse geographic regions, particularly in Europe, America, and areas with high amoxicillin resistance or widespread use of vonoprazan-based therapy. Prospective study registration, rigorous allocation concealment, and blinded study designs will help minimize bias. Further research is also needed to clarify strain-specific effects, dose–response relationships, and optimal treatment regimens to support evidence-based guideline recommendations.

## 5 Conclusion

Combining probiotics with BQT significantly improves the *H. pylori* eradication rate and reduces the adverse event rate. This finding supports the use of probiotics as a supplementary strategy for *H. pylori* eradication. However, the overall certainty of the evidence is low to very low, and the optimal probiotic protocol has yet to be determined, highlighting the need for further high-quality research.

## Data Availability

The original contributions presented in the study are included in the article/supplementary material; further inquiries can be directed to the corresponding author/s.
